# Supplementary material for: Are carbon emissions trading and green financial instruments synergistic? -Comprehensive quantitative research based on content analysis
Source: PLoS One. 2024 Mar 7;19(3):e0298601. doi: 10.1371/journal.pone.0298601 (PMC10919601; doi:10.1371/journal.pone.0298601)
Supplement: S1 Data — (ZIP) [file pone.0298601.s001.zip › Data/地级市Z_F工作报告.zip!/╦─┤¿/░═╓╨╩╨/└┤╫╘╣1⁄2╓┌║┼í╛┬φ┐╦╩2╛▌═°í┐_2014.docx]

各位代表：

我代表市人民政府向大会报告工作，请予审议，并请市政协委员和其他列席人员提出意见。

2013年工作回顾

过去一年，在省委、省政府和市委的坚强领导下，在市人大、市政协的监督支持下，我们认真贯彻落实党的十八大精神，牢牢把握科学发展、加快发展的总基调，着力统筹城乡、追赶跨越、加快发展，积极拼搏，扎实工作，经济社会发展取得新的成效。全市地区生产总值比上年增长10.7%，全社会固定资产投资增长35.4%，地方公共财政预算收入增长36.4%，社会消费品零售总额增长14.5%，城镇居民人均可支配收入增长11.4%，农民人均纯收入增长13.9%，城镇登记失业率4.3%。

投资实现快速增长。新开工重点项目148个，续建102个，完成投资681亿元，增长66.7%。交通、水利、能源等基础设施建设取得新的突破。成巴高速、巴达高速、巴汉高速巴城至南江北通车，巴广渝高速、巴达铁路加快建设，巴万高速开工在即，汉巴南铁路、广巴铁路技改扩能项目的前期工作有序推进。广巴、巴达、巴汉、巴广渝高速列入国家高速公路网。省道101线、202线、302线升为国道。巴中机场完成选址和预可研报告评审。红鱼洞和湾潭河水库开工，牛角坑、二郎庙、双桥和天星桥水库建设加快。巴中500千伏变电站及配套工程进展顺利，流坝220千伏和寨坡、驷马、笔山110千伏等输变电工程投入运营。新建成3G通信基站560个，农村光纤入户率达70%。

产业培育步伐加快。工业投资116.6亿元，增长52.1%。新引进5亿元以上工业项目12个，新开工61个，达芙妮鞋业等43个项目投产。新拓展工业园区5.1平方公里，巴中经济开发区列入全省“51025”重点产业园区发展计划。新增规模以上工业企业30家，规模以上工业增加值98.9亿元，增长11.2%。粮食稳定增产，全面启动山区现代农业示范市创建，新增现代农业万亩示范区12个、畜禽标准化养殖场（小区）56个、省级农业产业化龙头企业5家。实施农业品牌发展战略，推出“巴食巴适”综合性农产品区域公用品牌，获得“省级农产品产地无公害化市”命名。巴药发展行动计划扎实推进，新种植中药材2.5万亩，科创医药产业园开工。实施林业经济强市战略，启动茶叶、核桃双百工程，新增茶叶基地7.6万亩、核桃基地18.5万亩，新发展工业原料林13.5万亩、林下产业基地10.2万亩、林下养殖万亩示范片4个。完成12个重点镇、216个村土地承包经营权颁证试点工作，144个乡镇建立农村产权交易中心。服务业加快发展，中国（巴中）西部国际商贸城、盘兴中国西部建材物流园、平昌川东北国际商贸物流中心建设加快；新增限额以上流通企业107家；外贸进出口总额1.43亿美元，增长15%；光雾山成功创建“国家生态旅游示范区”，平昌县成功创建“全国休闲农业与乡村旅游示范县”，诺水河、佛头山4A级景区通过省级初检，启动光雾山国家5A级景区和“四川省旅游标准化示范市”创建，红叶节品牌效应增强，全年接待游客896.7万人（次），旅游收入66.2亿元；与13家省级金融机构签订战略合作协议，获授信710亿元，年内投放211.6亿元。辖区内银行机构存款余额678.6亿元、增长19.3%，贷款余额264.9亿元、增长30.1%。

城乡建设成效明显。坚持统筹城乡、产城一体，切实加强城乡规划建设管理。巴中市城市总体规划获得省政府批复，恩阳区城市控详规划正抓紧完善，4个县（区）完成全域规划，在全省率先实现市县（区）全域规划。完成城市规划区内4个镇总体规划、4个省级试点镇总规修编和18个重点镇、94个一般乡镇、70个中心村、963个聚居点规划。巴中经济开发区建设取得新的进展，建成骨架路网12公里，35公里市政道路顺利推进，招商中心、汽车客运中心、市第三水厂、城市规划馆等建成，市第一人民医院、兴文中学等加快建设，42万平方米安置还房即将建成交付。巴城麻柳湾大桥、南杨大桥、陇桥立交、中坝干道、巴恩快速通道西华山至燕飞村段建成通车，北环线形成通车能力，西环线加快建设，南环线开工，陇桥、回风、杨家坝、后河桥、莲花嘴、中坝等片区棚户区改造有序实施，新建巴城污水干管40公里，巴河一期堤防工程竣工。恩阳区新城建设全面启动，开工恩阳大道、恩阳新大桥、恩阳古镇保护开发等重点工程。平昌金宝、南江红塔、通江高明新区加快建设。启动4个省级试点镇建设，扎实推进市级重点镇建设。坚持产业先行、产村相融，推进以巴山新居为重点的新农村建设，启动新居建设4.37万户。扎实开展城乡建设领域“三违”整治，违法建设基本得到控制。深入实施“五创联动”和城乡环境综合治理，巴城成功创建省级森林城市、环境优美示范城市，南江、平昌、通江分别通过省级生态县、环保模范县、卫生县城验收。全市新增城市建成区面积10平方公里，城镇化率达到35.9%。

开放合作继续深化。积极扩大对外交往合作，与上海虹口区结为友好市区，加强与山东潍坊的城市战略合作。以产业招商为重点，扎实开展小分队招商、平台招商、以商招商。积极主动参与“央企入川、民企入川、港澳企业四川行”活动和第十二届华商大会、第十四届西博会，成功举办“对外开放·巴中行”、“光彩事业巴中行暨川商革命老区行”活动。全年签约项目198个，在建招商项目307个，年度到位资金286.49亿元，比上年增加92.52亿元。

民生事业持续改善。坚持优先发展教育，编制完成《农村义务教育学校布局专项规划》、《巴中市中心城区教育设施布点专项规划》，巴中职业技术学院建成招生，新建成中小学校舍24万平方米，改造农村寄宿制学校22所，全面实施学生“营养餐”计划。市中医院通过三级乙等综合医院评审，创建甲等乡镇卫生院33个、甲级村卫生室652个、标准化社区卫生服务中心8个，建立了大病保险、大病救助、门诊统筹和医疗纠纷第三方调解制度。全市千人病床数较上年增长12%。南龛文化产业园核心区加快建设，“米仓古道”申遗保护总体规划编制顺利推进，新建省级示范乡镇综合文化站17个、文化示范村253个。望王山运动公园对外开放，市体育馆、游泳馆项目加快推进，新增农民体育健身工程120个。与科研院所签订科技成果转化协议41项，申请专利326件，组织实施专利10件。人口自然增长率4.56‰。

坚持民生政府责任担当，深入实施省“十项民生工程”，认真办好市“十件民生实事”。建设乡镇联网路和断头路310公里、硬化村道路1600公里，新解决22.5万人的安全饮水问题。落实计划生育奖励扶助资金0.58亿元。城镇新增就业3.2万人，城乡居民养老、医疗保险参保人数分别达103.48万人、347.82万人，覆盖率分别达到85.7%和97.3%，城乡一体的社会保障体系基本形成，城市、农村低保对象月人均补助标准分别提高到195元和93元，集中供养、分散供养五保对象月人均补助标准分别提高到350元和250元。城乡困难群众住院救助力度加大。开工建设公共租赁住房805套，启动改造各类棚户区2.56万户，基本建成1.05万户，竣工8330户。完成农村危旧房（土坯房）改造4.67万户，建设拆迁安置还房417万平方米，处理房屋“两证”遗留问题120万平方米。切实抓好巴城“菜篮子”工程，新建蔬菜基地1.6万亩。新启动12个片区连片扶贫开发，实施扶贫整村推进项目63个，全市减少贫困人口5.76万人。

社会保持和谐稳定。创新社会治理方式，加强乡镇（街道）和社区便民服务中心建设，新建农村社区60个，建成农村社区服务中心200个、城镇社区服务中心60个，规范194个乡镇（街道）便民服务中心运行，34项行政审批项目、101项公共服务事项下放到乡镇（街道）和社区便民服务中心。牢固树立底线思维，落实“一岗双责”，抓好信访安全稳定工作。实施社会稳定风险评估制度，集中化解了一批老难信访件。强化安全隐患排查、整改，全面落实操作者责任。推进煤矿企业清理整顿关闭重组，关闭煤矿8家，超省政府目标2家。实施重大地质灾害治理工程61处，完成地质灾害避让搬迁安置3974户，实现汛期地质灾害“零伤亡”。全市安全生产形势持续稳定，无重特大安全事故发生。完成食品、药品安全监管职能整合，建立健全食品药品安全监管体系，严厉打击食品药品安全违法犯罪行为。开展娱乐场所专项整治“百日会战”，全面整治巴城126家娱乐场所，关闭3家，立案查处网吧14家，查禁涉赌电子游戏场所37家。着力抓好天网工程升级改造、行业场所实名制管理、城乡警务运行机制和户籍管理制度改革试点等工作，打击违法犯罪行为，社会治安秩序良好。

老龄、妇女儿童、残疾人等事业取得新的进步，国防动员、民兵预备役、双拥、人防、档案、地方志、民族、宗教、侨务、外事、对台、保密、地震、气象等工作取得新的成效。

我们注重加强政府自身建设，修改完善《政府工作规则》，建立政府决策法律顾问组，着重提高依法行政水平和行政效能。完成行政审批事项第十一次清理，公共资源交易逐步规范。高度重视办理人大代表建议和政协委员提案，努力解决群众高度关注和反映迫切的问题。认真学习习近平总书记系列重要讲话精神，扎实开展“实现伟大中国梦、建设美丽繁荣和谐四川”主题教育活动和“转作风·惠民生”群众路线教育实践活动，严格执行改进作风密切联系群众和厉行勤俭节约、反对铺张浪费等各项规定，坚决反对“四风”。行政监察和审计监督得到加强，惩防体系进一步健全，廉政建设取得新的进展。

这些成绩的取得是省委、省政府和市委坚强领导，人大代表、政协委员监督支持，全市干部群众团结奋斗的结果。在此，我代表市人民政府，向全市干部群众，向驻巴部队、武警官兵和政法干警，向各民主党派、工商联、无党派人士、各人民团体，向关心和支持巴中发展的社会各界朋友，表示崇高的敬意和衷心的感谢！

我们也清醒地看到，在宏观经济形势极为错综复杂的背景下，地区生产总值、社会消费品零售总额等指标与年初预期目标还有差距；要素支撑能力偏弱，产业培育进度不快；边远山区群众生产生活条件还比较艰苦；一些政府工作人员驾驭市场经济的本领不强，法治思维、履职尽责意识和改革创新能力还有待提升。我们一定高度重视群众的关切，直面自身工作中的问题，采取更有针对性和实效性的措施认真加以解决。

2014年主要任务

2014年，我们要认真贯彻党的十八届三中全会、中央省委经济工作会议及市委三届七次全会精神，坚定不移地实施“三大发展战略”，坚持“统筹城乡、追赶跨越、加快发展”的总体要求，紧盯发展目标，专注发展定力，坚持问题导向，以改革开放、扶贫攻坚为统领，强化产业支撑、投资拉动、消费带动、民生改善，全力巩固和扩大追赶跨越发展态势。

综合考虑各种因素，今年全市经济社会发展主要预期目标是：地区生产总值增长11%，全社会固定资产投资增长30%，规模以上工业增加值增长12%，社会消费品零售总额增长15%，地方公共财政预算收入增长20%，城镇居民人均可支配收入增长13%，农民人均纯收入增长14%，城镇化率提高2.2个百分点，城镇登记失业率控制在4.2%以内，森林覆盖率达到57.1%，单位生产总值能耗下降2个百分点。

实现上述目标，要重点抓好以下七个方面工作：

一、全面深化改革开放

推进重点领域改革。今年是全面深化改革年，政府各方面工作都要以改革为统领，把改革贯穿于经济社会发展的各个领域和各个环节，按照中央和省委、市委的决策部署，有序推进重点领域的改革。推进农村综合改革，加快农村土地承包经营权确权颁证，建立健全农村产权交易市场，促进农村“八权一股”自由平等有序流转，增加农民财产性收入。深化城乡建设用地增减挂钩，建立农村宅基地自愿有偿退出机制，推动农村集体土地公司化、股权化、股份化改革，切实解决已转移到城镇就业的农业转移人口的落户问题，保留农业转移人口原农村承包地、林地等权益。推进市场化改革，全面落实负面清单及先照后证、注册资本金认缴登记等制度，完善民间投资激励扶持政策措施，畅通民间资本参与基础设施建设和公共事业、社会事业的发展渠道。鼓励非公有制企业参与国有企业改革，大力发展混合所有制企业。实施科技创新驱动，鼓励企业与科研院所、高等院校建立产学研联盟，推动科研成果转化利用。推进财税体制改革，健全事权与支出责任相适应的制度，完善公共财政体系，创新绩效管理，提高财政资金的使用效益。深化教育、文化、卫生等领域改革，鼓励支持社会组织发展，探索政府购买公共服务。

扩大充分开放合作。开放是加快老区发展的主要推手，也是更深层次的改革。积极参与国家、省组织的各类投资促进活动，瞄准国际国内大企业、大集团，深度开展产业链招商、小分队招商和点对点招商。强化跟进对接和跟踪服务，推动招商引资项目落实落地，实现招商引资到位资金330亿元以上，增长15%。强化多层次多区域合作，巩固市厅、市校、市院战略协作关系，深化与泉州、虹口、烟台等友好市（区）在承接产业和资本转移方面的合作机制，加强与周边市在重大基础设施建设、产业培育、环线旅游以及秦巴山片区中心城市的政策争取、重大项目包装、打造国家山地森林公园共同体等方面的协同合作。加强对优势潜力企业帮扶指导，鼓励拓展国际市场，外贸出口增长8%。

二、强化项目投资拉动

加强项目建设。发挥投资在稳定增长和形成生产能力方面的双重作用，以项目工作为总抓手，计划实施重点项目238个，年度完成投资760亿元。瞄准国家和省重大政策导向，积极争取国家出台川陕苏区振兴发展规划，全力争取川陕苏区核心区域的特殊政策，紧紧抓住省委省政府支持川东北经济区、革命老区加快发展的政策机遇，编制储备一批关系巴中长远发展的重大项目。全面实施《秦巴山片区区域发展与扶贫攻坚规划》及省级实施规划，促进规划项目尽快启动实施。推进重点项目建设，新开工巴万高速公路等112个重点项目，提速建设红鱼洞水库等121个续建项目，力争省道大佛寺至兴文过境公路等37个重点项目竣工投产。加快广巴铁路技改扩能和黄石盘、青峪口、江家口水库等项目的前期工作。推进重大基础设施建设，争取汉巴南铁路纳入国家《中长期铁路网规划（中期调整）》，建好成巴、巴达、巴汉高速服务区等配套设施，加快巴汉高速南江至桃园段、巴广渝高速建设进度。完成巴达铁路轨道铺设及部分线上工程，力争巴中机场开工建设。完成258公里国道改造，力争部分县乡公路纳入省道公路规划。加快天星桥、湾潭河等水库建设。竣工投运巴中500千伏输变电工程，抓好张公220千伏等输变电工程建设。

强化要素保障。加快组卷报征建设用地进度，加强土地整理储备，将巴山新居工程区域纳入城乡建设用地增减挂钩项目范围，强化重点项目用地保障。采取整合国有经营性资产直接注入或划转股权注入等方式，做大做强国有投融资平台，加快启动国企第二期债券发行工作，提升市、县（区）投融资能力。加大与政策性银行对接力度，加快省级银行机构710亿元授信投放，推行工业集中发展区整体授信、企业抱团互联互保和统贷统还等融资模式，提高银行存贷比，强化重点项目融资保障。积极组织各项收入，认真筹措项目回购资金，缓解重点项目资金压力。加快水、电、气、通讯等设施建设，强化重点项目基础配套保障。加强投资软环境建设，建立“开放竞争力评价”体系，营造与开放型经济相适应的政务环境和法治环境。建立专班负责项目跟进、专题研究项目难题、专人联系项目信息的全程跟踪服务机制，确保项目建设顺利推进。

三、推进产业加快发展

着力壮大工业实力。制订和实施巴中经济开发区500亿产业园、平昌100亿产业园、其他县（区）50亿产业园的四年推进计划，拓展园区面积3.5平方公里。加快产业园区基础设施配套建设，积极引进和培育龙头企业。支持兆润摩托、达芙妮制鞋等企业扩能达产。加快海螺水泥、万马汽车、同凯能源、建丰新材等在建项目建设进度，尽快投产达效。新增20户规上企业，实现规上工业增加值110亿元。用好用活工业发展引导资金，采取财政贴息和贡献奖励等方式加大扶持力度。推进技术创新和科技成果转化，完成技改投资70亿元。

积极培育现代农业。开展山水田林路综合整治，加强农田水利基本建设，夯实现代农业发展基础，稳定粮食生产。突出绿色生态富硒特色，着力创建山区现代农业示范市，新建12个现代农业万亩示范区。推进农产品精深加工，制订银耳、川明参、芦笋等优势产品的国家、省级生产技术标准，做强“巴食巴适”综合性农产品区域公用品牌。深入实施巴药发展行动计划，加强巴药基地建设，加快龙头企业培育。全面实施林业经济强市战略，培育“4＋1”林业支柱产业，全市林业总产值达到120亿元。创建山区现代畜牧业示范市，依托温氏集团带动，新增出栏生猪20万头以上；支持新农村标准化养殖场（小区）建设，加强地方畜禽品种选育保种，着力推动林下养殖示范片建设。构建现代农业经营体系，支持农民发展专业合作、股份合作、社区合作等农村合作经济；鼓励引导工商和社会资本投向农业农村建设，支持农民合作社开展信用合作试点。

推进服务业集聚发展。发挥生态旅游资源优势，重点发展山地休闲度假旅游、城镇人文观光旅游及特色乡村生态旅游，完善全域旅游业发展规划，加强产业融合，引进战略合作伙伴深度开发旅游资源，完善旅游基础设施，创建全省旅游标准化示范市和一批国家、省级旅游景区，加强旅游宣传营销，提升区域旅游经济综合竞争力，实现旅游收入75亿元以上。推进川陕渝商贸物流枢纽建设，加快中国（巴中）西部国际商贸城、盘兴中国西部建材物流园建设，促进商贸物流业集聚集群发展。新建、改建10条特色商业街区，加快实施“新网工程”。积极培育文化娱乐、健康服务等热点消费和家政、物业等便民消费。新增50家限额以上流通企业，外贸进出口总额实现新突破。加快培育金融业，争取列为全省农村金融改革试点，集中精力抓好民间资本设立民营银行、金融租赁公司和消费金融公司工作，引进（设立）各类金融机构10家。创建金融生态环境暨金融安全发展示范县（区），优化信贷结构，提高金融服务能力。

四、提高城镇建设水平

统筹推进城乡建设。强化规划引领，着力提质扩容，优化城乡空间形态，巴城主城区建成区面积新增6平方公里。中心城区启动山地城市公园、山地森林公园建设，抓好绿化、亮化景观工程，积极推进回风、陇桥、北龛寺、中坝等城市拓展区建设，有序推进旧城改造升级。加快巴中经济开发区核心区建设，全面完成路网工程，有序启动清江、奇章片区快速通道和拓展片区骨架路网建设，完善市政功能配套，市第一人民医院、城市综合体等项目建成运行，启动6.6平方公里山地运动公园建设，加快推进湿地公园、河道景观等工程，基本形成新区“一心两幅三片多点”城市景观体系。加快恩阳新城建设，完成地下空间、古镇保护等专项规划和重点区域详规，加快建设巴恩大道、恩阳新大桥等城市基础设施和学校、医院等社会事业项目，推进巴城西翼快速发展。以中等城市标准加快三县城建设。着力抓好诺水河镇等8个省级试点镇和驷马镇等9个市级重点镇，有序推进一般集镇建设。深入推进巴山新居工程，充分尊重群众意愿，发挥主体作用，突出怡居宜业，坚持产业先行、产村相融，合理定位新村形态和业态，建设中心村22个、聚居点310个。深化“五创联动”工作，推进国家卫生城市、森林城市、环保模范城市和省级生态市创建。

加强配套功能建设。着力构建规划衔接、基础完善、功能配套的市政设施体系。抓好中心城区绕城环线、江南二环路、东门大桥等城市交通项目，提高城市路网密度，建立干支衔接、内畅外达的交通网络。全面启动建设城镇地下综合管廊管沟，推进供电、供水、供气和通信管线地下化。加快完善江南片区停车场等城市配套功能。完善城镇防洪设施建设，提高城镇防洪能力。加快城市建成区、产业园区、小城镇和新型农村社区的污水、垃圾处理设施建设。严格城镇规划管理，优化城镇功能分区，增加居民居住、休闲场所等用地，切实提高城镇建设用地集约化程度。严格保护城镇人文景观、特色风貌和生态环境，严厉整治违法建设，严禁无序开发和破坏性建设，努力提升城镇品质。

五、深入实施民生工程

提升民生保障水平。落实各项就业优惠政策，加强就业技能培训，拓宽就业渠道，积极开发公益性岗位，全力做好城镇就业困难人员、农村转移劳动力、高校毕业生等就业工作，实现城镇新增就业2.6万人，鼓励引导在外成功人士回乡创业，扶持新增创业1000户。完善统筹城乡养老、医疗、失业、工伤、生育保险制度，探索建立城镇职工“五险合一”的征缴机制，改进医疗保险付费方式，强化社会保险扩面征缴工作，确保城乡养老保险参保率达95%、基本医疗保险参保率达97.3%以上。完善社会救助体系，支持发展慈善事业。

推进连片扶贫开发。新启动覆盖15个片区、19个乡镇、215个行政村、28.7万人的连片扶贫开发，减少贫困人口4.7万人以上。大力实施“六大扶贫工程”，突出解决连片特困地区行路、饮水、上学、就医和住房安全问题，加快资源开发和特色优势产业培育，增强发展造血功能。有序实施边远高寒山区生态移民工程。

切实办好民生实事。认真实施省“十项民生工程”，办好市“十件民生实事”。解决住有所居问题，深入推进“农民工住房保障行动”，新开工建设公共租赁住房8180套，改造各类棚户区3.45万户、农村危旧房（土坯房）4.8万户，推进拆迁安置还房建设，避险搬迁安置1000户。抓好乡村道路工程，实施通村通畅工程760公里、农村公路改善工程230公里。开展关爱留守儿童、留守老人行动，扎实做好困难群众救助工作，确保长期大病患者、精神病患者、重度残疾人全部纳入城乡低保。落实“菜篮子”工程行政首长负责制，新增商品蔬菜基地1.5万亩，提高蔬菜自给能力。加强生态文明建设，坚持节约优先、保护优先，增强生态产品生产能力和优良环境保护能力，实施“碧水蓝天绿地”工程，强化环境监测，加强节能减排和面源污染治理。抓好巴城第二水源化成水库、巴城上游水资源和5县（区）城区饮用水源地保护，完成188个乡镇集中式饮用水源保护区划定，健全水质监测体系，新解决农村12万人安全饮水问题。实施农村低电压改造工程，切实保障电力稳定供给。

六、切实加强社会建设

促进教育均衡发展。优化城乡校点布局，有效整合教育资源，加快改善办学条件，建设农村义务教育寄宿制学校35所，新建、改扩建校舍20万平方米。实施教育信息化建设工程，推动教育开放合作，引进培育扩大优质教育资源。加强教师队伍建设，建立市县校三级培训网络，落实城乡教师定期交流制度，提高村小教师待遇。完善四级职教体系，实施五大培训工程，发挥巴中职业技术学院龙头引领作用，促进产教融合。加快棠湖外语实验学校、川师大附属第四实验中学等民办学校建设，促进公办、民办教育协调发展。

提升医疗服务能力。健全卫生服务体系，新开工市传染病医院、全科医生临床培养基地、儿童医院等卫生项目建设，市中心医院创建三级甲等综合医院，启动县（区）医院三级乙等综合医院创建，建设甲等乡镇卫生院15家、标准化社区卫生服务中心4家、村卫生室200个。抓好卫生专业技术人才引进和培训，扩大乡村卫生“一体化管理”范围，全面推进乡村医生签约服务。完善中医诊所基础设施，提升基层中医药服务能力。积极实施“单独两孩”政策。

发展文化体育事业。加快市、县（区）、乡（镇）、村（社区）文化阵地建设，构建全域覆盖、优质高效的公共文化服务体系。实施文化精品工程，开展送文化下乡活动，抓好广播电视安全播出，提升“秦巴大讲堂”等公共文化服务品牌，努力丰富群众文化生活。加大“米仓古道”申遗力度，加强文化遗产保护。建立健全长效机制，加强文化市场监管。完成南龛文化产业园核心区建设，加快佛头山、恩阳古镇、晏阳初等文化产业园建设，带动文化企业、文化人才等文化要素向园区聚集。建成市体育馆、游泳馆和50个农民健身工程，大力开展群众体育活动。

提升社会治理水平。健全基层综合服务管理平台，及时反映协调各方面各层次利益诉求，积极引导群众平等参与自治和管理，改进社会治理方式。加强普法教育，弘扬法治精神。建立完善网上受理信访制度，畅通和规范群众诉求表达渠道。深入开展“平安村居”、“平安小区”创建活动，依法严厉打击各类违法犯罪活动。实行食品药品安全全过程监管、原产地可追溯和质量标识制度。加大矿山、道路交通、建筑施工、危化品等重点行业、重点领域安全生产标准化建设和专项整治力度，认真开展道路交通安全专项整治攻坚年行动。完善应急救援机制，预防和处置好各类突发事件。

七、推进政府自身建设

建设法治政府。加快法治巴中建设，提高依法行政水平。自觉把合法作为行政决策的第一要件，坚持用法治思维和法治方式处理问题、推动工作，健全重大事项集体决策、开放式决策、专家咨询论证、社会稳定风险评估、合法性廉洁性审查和公开听证制度，严格按照法定权限和程序行使权力、履行职责。规范行政执法行为、程序和自由裁量权，做到严格执法、公正执法、文明执法。自觉接受市人大及其常委会的法律监督和工作监督、市政协的民主监督、新闻舆论监督、社会公众监督，认真听取民主党派、工商联、无党派人士的意见，加强监察、审计等内部监督，确保行政权力依法、公正、有效运行。

建设服务政府。强化履职尽责意识，围绕政府工作主要任务完善分工负责、合力推进机制，严格落实工作责任制和项目负责制，开展政府部门履职评估，落实行政问责制，大力整治庸懒散，切实提高执行力。以自我革命的精神切实转变政府职能，处理好政府与市场、政府与社会的关系，努力为市场主体创造更加公平有序的发展环境。深入开展党的群众路线教育实践和“转作风·惠民生”活动，努力为广大群众排忧解难，积极适应新型城镇化进程加快、群众需求日益多元的新形势，把工作着力点转变到服务基层、企业和群众上，实现乡村便民服务站点全覆盖，面向群众服务的事项全部纳入便民服务中心窗口办理，面向企业的行政审批全部纳入政务服务中心办理。整合政务服务和公共资源交易平台建设，推进并联审批、网上审批，切实提高行政效率。

建设廉洁政府。推行行政权力清单制度，坚持以公开为原则、不公开为例外，利用政务服务中心、公共资源交易服务中心、政府门户网站等平台，依法依规公开重大决策、重大投资、行政审批事项。严格落实厉行节约、严禁铺张浪费的规定，艰苦创业、勤俭办事，降低行政成本，积极化解政府债务。严格落实廉政建设责任制和领导干部廉洁自律各项规定，坚持用制度管权、管事、管人。严格落实工程建设招投标、经营性土地招拍挂、政府采购、国有产权转让、特许经营权出让等相关制度，深入推进工程建设领域专项治理。加强廉政文化建设和廉政教育，严厉查处各类违法违纪案件，坚决纠正损害人民群众利益的不正之风。

各位代表，巴中发展，任重道远，时不我待。让我们紧密团结在以习近平同志为总书记的党中央周围，在省委、省政府和市委的领导下，群策群力，开拓创新，为推动巴中追赶跨越、加快发展而努力奋斗！
